# How Does Ionizing Irradiation Contribute to the Induction of Anti-Tumor Immunity?

**DOI:** 10.3389/fonc.2012.00075

**Published:** 2012-07-25

**Authors:** Yvonne Rubner, Roland Wunderlich, Paul-Friedrich Rühle, Lorenz Kulzer, Nina Werthmöller, Benjamin Frey, Eva-Maria Weiss, Ludwig Keilholz, Rainer Fietkau, Udo S. Gaipl

**Affiliations:** ^1^Radiation Immunobiology, Department of Radiation Oncology, University Hospital Erlangen, Friedrich-Alexander Universität Erlangen-NürnbergErlangen, Germany; ^2^Department of Radiotherapy, Clinical Center BayreuthBayreuth, Germany

**Keywords:** low and high dose ionizing irradiation, immune modulation, immunogenic cancer cell death, dendritic cells, abscopal effects, immune therapy, AnnexinA5, hyperthermia

## Abstract

Radiotherapy (RT) with ionizing irradiation is commonly used to locally attack tumors. It induces a stop of cancer cell proliferation and finally leads to tumor cell death. During the last years it has become more and more evident that besides a timely and locally restricted radiation-induced immune suppression, a specific immune activation against the tumor and its metastases is achievable by rendering the tumor cells visible for immune attack. The immune system is involved in tumor control and we here outline how RT induces anti-inflammation when applied in low doses and contributes in higher doses to the induction of anti-tumor immunity. We especially focus on how local irradiation induces abscopal effects. The latter are partly mediated by a systemic activation of the immune system against the individual tumor cells. Dendritic cells are the key players in the initiation and regulation of adaptive anti-tumor immune responses. They have to take up tumor antigens and consecutively present tumor peptides in the presence of appropriate co-stimulation. We review how combinations of RT with further immune stimulators such as AnnexinA5 and hyperthermia foster the dendritic cell-mediated induction of anti-tumor immune responses and present reasonable combination schemes of standard tumor therapies with immune therapies. It can be concluded that RT leads to targeted killing of the tumor cells and additionally induces non-targeted systemic immune effects. Multimodal tumor treatments should therefore tend to induce immunogenic tumor cell death forms within a tumor microenvironment that stimulates immune cells.

## Introduction

The old theory about immunological tumor control has been revived in the last decades. Especially preclinical experiments with immune deficient mice coined the immune editing hypothesis of cancer. Recombinase-activating gene 2 (RAG2) deficient mice lack functional T and B cell receptors and develop tumors more quickly and with greater frequency than immune competent wild-type mice (Shankaran et al., 2001). The cancer immune editing model provides a well reflected explanation regarding how cancer cells get eliminated by the immune system, stay calm (equilibrium phase), or escape immune surveillance. This knowledge is a valuable basis for the design of immunotherapies against cancer (Vesely et al., 2011).

Clinical studies evaluating the frequency of cancer in immune suppressed patients further support that the immune system is involved in tumor control (summarized in Mueller, 1999; Zitvogel et al., 2010). Changes in the surface expression of major histocompatibility complex (MHC) I molecules have a major impact on the prognosis of tumor patients. Since only the tumor peptide/MHC I complexes are recognized by cytotoxic T lymphocytes (CTLs), the contribution of the immune system to a successful cancer therapy has become evident. Importantly, the surface modifications of tumor cells result from the selection pressure (immune editing) exerted also by cells from the innate and adaptive immune system (Pages and Kroemer, 2011).

Nowadays the pivotal question is not whether the immune system contributes to tumor control but rather in which phase of tumor disease and after which treatment combinations. Since immune cells cannot cope with big tumor masses, additional therapies are needed to reduce the tumor volume or to render the cancer cells visible for immune attack. Certain chemotherapeutic agents (CT) and ionizing irradiation (X-ray) that is applied in radiotherapy (RT), mostly in combination with further immune stimulation, may render the tumor cells immunogenic. We assume that low and high doses of X-ray modulate the immune system and focus on abscopal anti-tumor immune responses that are induced by combinations of standard tumor with immune therapies.

## Immune Modulation by Low and Intermediate Dose Radiation

Exposure to radiation always has been a point of concern for many people, especially in times when nuclear power plant accidents occur. Radiation is often associated with being a threat to humans causing cancer and other diseases. Another rising source of radiation is medical applications. The latter increased the total effective collective dose of irradiation to humans by 70% over the last years [United Nations Scientific Committee on the Effects of Atomic Radiation (UNSCEAR) Report 2008]. Fortunately, the effects of radiation on the immune system being a first line defense system against malignancy have attracted the notice of researchers and clinicians.

### Low dose radiotherapy induces anti-inflammation – the role of macrophages

One should distinguish between high dose (single-dose >1.0 Gy), intermediate dose (single-dose >0.1 and ≤1.0 Gy), and low dose radiation (≤0.1 Gy; Salomaa et al., 2010). Low and intermediate dose radiation (low dose RT, LDR) is used to treat acute and chronic painful inflammatory diseases. LDR induces anti-inflammation by, e.g., hampering leukocyte adhesion to endothelial cells (ECs), induction of apoptosis, reducing the activity of the inducible nitric oxide synthase, and by lowering the oxidative burst in macrophages (summarized in Rodel et al., 2012).

Monocytes and macrophages are key players in initiation, maintenance, and resolution of inflammation (Fujihara et al., 2003; Hume, 2006; Valledor et al., 2010). They support the inflammatory host response by secreting pro-inflammatory cytokines such as tumor necrosis factor (TNF)α, interleukin (IL)-1β, and IL-6. The immune response is further amplified by release of reactive oxygen species (ROS) and nitric oxide (NO). On the other hand they may initiate the healing process and are involved in resolution of inflammation by phagocytosis of apoptotic cells and cell residues as well as by secreting anti-inflammatory cytokines such as IL-10 and transforming growth factor (TGF)β (Martin and Leibovich, 2005; Anders and Ryu, 2011). Inappropriate regulation of the resolution process can result in severe chronic inflammation and autoimmune diseases. Improvements of inflammatory diseases and pain after LDR have been observed in patients for over 100 years (summarized in Kern et al., 1999). This suggests that macrophage-mediated modulations of inflammation can be influenced by LDR.

Macrophages are considered to be radio-resistant while monocytes are more sensitive to radiation (Hildebrandt et al., 1998; Bauer et al., 2011). Monocytes are impaired in DNA double-strand break (DSB) repair; however even their apoptotic rate merely increased up to 10% following LDR (≤1.0 Gy). It therefore can be assumed that the anti-inflammatory effects of LDR are not caused by dying phagocytes themselves (Voll et al., 1997), but rather by regulatory mechanisms. Discontinuous dose dependence with local peaks within a dose range of 0.3–0.7 Gy has been observed in many assay systems where macrophages were exposed to LDR.

X-ray treatment with single-doses between 0.3 and 0.6 Gy reduces the production of ROS by activated macrophages. ROS enhances the destruction of pathogens, but could also lead to serious destructions of own tissue, if deregulated like in chronic inflammation and autoimmune diseases (Schaue et al., 2002). Further, a reduced activity of the inducible Nitric Oxide Synthase (iNOS) and a lowered concentration of its immune regulatory product NO take place in activated macrophages following LDR. The changes occurred on protein and not on mRNA level (Hildebrandt et al., 1998, 2003; Rodel et al., 2002). NO is a key mediator of cytotoxic and immune stimulating effects. It is produced and secreted by inflammatory macrophages. A significant inhibition of NO production in macrophages was observed after LDR, while X-rays with doses ≥5 Gy increased it (Hildebrandt et al., 1998, 2003). Since NO influences the expression of inflammatory cytokines (Abramson et al., 2001), it may serve as a link between LDR and inflammatory cytokine expression.

Decreased levels of the pro-inflammatory cytokine TNFα were measured when Lipopolysaccharide (LPS)-activated macrophages were irradiated with 0.5 or 0.7 Gy of X-rays (Tsukimoto et al., 2009; Rodel et al., 2012). An involvement of the ERK1/2 and p38-MAPK pathways in triggering such anti-inflammatory responses is likely. Both pathways are deactivated by dephosphorylation via the protein phosphatase MKP-1. Tsukimoto et al. (2009) reported that 0.5 Gy of γ-irradiation significantly increases the expression of MKP-1, inactivates p38-MAPK, and finally suppresses the TNFα production in mouse RAW264.7 macrophages. Actually many of such anti-inflammatory properties of LDR are regulated by nuclear factor kappa-light-chain-enhancer of activated B cells (NFκB) on a transcriptional level. A reduced translocation of NFκB into the nucleus has been observed in various inflammation models after exposure to LDR (summarized in Rodel et al., 2012).

The amount and nature of cytokines which are produced and released by macrophages following LDR also depend on the presence of dying cells in the microenvironment. Activated monocytes/macrophages secrete anti-inflammatory cytokines (IL-10, TGFβ), rather than pro-inflammatory ones (IL-1β, TNFα) in the presence of apoptotic cells (Voll et al., 1997). The apoptotic rate of peripheral blood mononuclear cells (PBMCs) increased following LDR with a maximum in the dose range of 0.3–0.7 Gy (Kern et al., 1999). The clearance of such apoptotic cells and/or cell residues is predominantly carried out by macrophages. More information on how LDR influences the phagocytosis of apoptotic and necrotic cells is urgently needed. First investigations with latex beads as prey revealed that low dose X-irradiation of LPS-activated macrophages reduces the phagocytosis. In contrast, higher single-doses (≥5 Gy) slightly increase the uptake of beads by activated macrophages (Conrad et al., 2009). The phagocytosis of colorectal tumor cells by macrophages and dendritic cells was shown to be reduced when the tumor cells (and not the macrophages) had been irradiated with higher doses of X-ray (2, 5, or 10 Gy). It should be stressed that the phagocytosis can be significantly enhanced when X-ray is combined with heat treatment (hyperthermia) of the tumor cells (Schildkopf et al., 2011).

Such impacts of LDR on macrophages, displaying repeatedly local peaks in a dose range of 0.3–0.7 Gy, could be a consequence of one central process affected by LDR. Also in other immune cells such as PBMCs, polymorphonuclear cells (PMNs), and ECs similar immune modulations induced by LDR have been observed, including a discontinuous dose-dependent translocation of NFκB into the nucleus (Prasad et al., 1994, 1995; Kern et al., 1999; Roedel et al., 2002;Rodel et al., 2004a,b, 2009; Gaipl et al., 2009). Since NFκB is a key transcription factor for a variety of immune factors such as cytokines, adhesion molecules, and growth factors and additionally is a potent post-transcriptional regulator of iNOS (Vodovotz and Bogdan, 1994), its modulation may therefore play a prominent role in the induction of an anti-inflammatory response following LDR. We have recently reported that a reduced secretion of IL-1β by stimulated macrophages after exposure to LDR correlates with a reduced nuclear translocation of p65 (RelA) of the NFκB-complex (Lodermann et al., 2012). Nevertheless, LDR has been shown in experimental animal models to temporarily suppress immune functions by a variety of other mechanisms. Examples are the disturbance of cells of the cellular and humoral immunity or the reduction of the viability of mature blood cells by affecting the hematopoiesis (Yagunov et al., 1998; Serhatlioglu et al., 2004).

### LDR stimulates immune functions

Other experiments link chronic and acute irradiation with low and intermediate doses with an enhanced immune function (Liu et al., 1987; James and Makinodan, 1988; Liu, 2007). Liu and colleagues showed that a variety of immune functions are stimulated by LDR such as natural killer (NK) cell and macrophage activity, or proliferation of T cells. Another feature of chronic exposure to LDR is the induction of an altered cytokine profile in the peripheral blood that can arise from activation of innate immune responses and not from changing the total number of white blood cells, red blood cells, and platelets (Shin et al., 2010).

The enhanced immune functions induced by LDR could explain why whole body LDR exposure of mice can reduce tumor outgrowth of B16 melanoma and Lewis lung cancer as well as metastasis formation after tumor cell inoculation (Hosoi and Sakamoto, 1993; Liu, 2003). Furthermore, the carcinogenic effect of high dose irradiation can be suppressed by a previous whole body irradiation with a dose of 0.075 Gy in C57BL/6 mice to a certain extent, mostly likely due to LDR-induced immune activation against tumor cells (Ina et al., 2005). These findings could shed some light on why people who are exposed to LDR by a higher background of earth radiation or through work situation display a decreased incidence for certain cancers or an elevated life span. This hypothesis is further supported by epidemiological studies, such as the British nuclear workers 51 year study (McGeoghegan and Binks, 2001; Atkinson et al., 2004) or the Hanford downwind inhabitants 50 years’ survey (Boice et al., 2006).

The knowledge that LDR also activates immune functions is not only helpful for radiation safety questions and associated guidelines, but also for clinical applications where cancer patients are treated with high dose radiation therapy (RT). A hint that whole body irradiation with LDR could improve the effects of standard RT is provided by animal studies of Jin and colleagues. They compared a fractionated local RT of 6 × 5 Gy of Lewis lung cancer in C57BL/6 mice with a modified fractionated RT in which the second/fifth and third/sixth fraction of the locally applied irradiation with 5 Gy was substituted by a whole body irradiation with 0.075 Gy (Jin et al., 2007). Since the tumor outgrowth reflecting the therapeutic effect was comparable in both schemes, the total irradiation dose could be reduced by two-third when LDR was included. In another experiment, a radiation scheme of 6 × 2 Gy over 2 weeks was compared with a local 2 Gy irradiation and a double administrated whole body irradiation with 0.075 Gy, which was given twice in the same time frame [2×(2 Gy + 0.075 Gy × 2)]. In this case, a significant slower tumor outgrowth in the whole body irradiated group of mice was observed, although the total dose was reduced to one-third (Jin et al., 2007).

## Immune Activation by High Dose Radiation

The immune stimulating potential of high dose radiation does initially not appear obvious since RT induces a time-restricted immune suppression by directly destroying immune cells (Anderson and Warner, 1976). However, in contrast to CT, the immune suppressive effects of RT are lower and more localized (Hodge et al., 2008). Nevertheless, CT and RT can both signal to the immune system tumor cells that had previously escaped immune surveillance (Ma et al., 2010). The phenotype of cancer cells has to be modified by therapeutic tools in a way that immune cells are attracted, induced to mature, and activated. For example, RT enhances the degradation of existing proteins inside the cells and also concomitantly the surface expression of MHC class I molecules (Reits et al., 2006). Complexes of MHC I molecules with peptides are recognized by CTLs that specifically kill tumor cells. RT further promotes the priming of antigen-specific DCs (Lee et al., 2009) and may increase the number of antigen presenting cells within tumor-draining lymph nodes (LN; Lugade et al., 2005) where antigen presentation by DCs and activation of CD8+ CTLs takes place.

### Immunogenic tumor cell death

**Figure 1:**
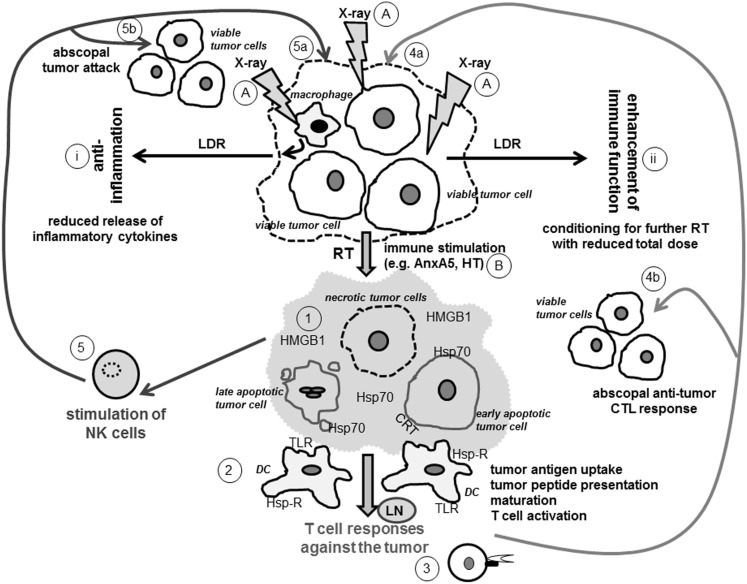
**Ionizing radiation modifies the tumor cell phenotype and induces a tumor microenvironment that fosters innate and adaptive immune responses against the tumor**. Viable tumor cells or cells of the tumor microenvironment, as exemplarily displayed here for macrophages, modify their phenotype when exposed to X-rays (A). LDR leads to reduced secretion of inflammatory cytokines by macrophages and induces an anti-inflammatory environment (i). It additionally conditions the tumor for further treatment with higher doses of X-ray (ii). The latter are applied in RT as part of cancer treatment and induce distinct forms of tumor cell death (1). Early apoptotic tumor cells expose CRT, a recognition molecule for their phagocytosis by DCs. Late apoptotic cells release blebs that carry danger signals such as Hsp70 and thereby activate DCs. Necrotic tumor cells have lost their membrane integrity and therefore release immune activating danger signals (e.g., HMGB1 and Hsp70) that interact with receptors such as TLR and Hsp-R on DCs. Necrotic cells might also directly be induced by combination of stress stimuli such as RT and HT (B) or be secondary necrotic ones when the uptake of apoptotic tumor cells by macrophages is blocked with AnxA5 (B). The distinct forms of dying and dead tumor cells create an immune stimulatory tumor microenvironment (displayed in light gray) that fosters tumor antigen uptake and maturation of DCs (2). The latter process tumor antigens and (cross-) present tumor peptides via MHC class I and class II molecules to CD8+ and CD4+ T cells, respectively (3). CD8+ CTLs recognize tumor peptides presented in MHC class I molecules on tumor cells and specifically attack therapy-modified tumor cells (4a) as wells as non-treated ones (4b). The tumor microenvironment and the tumor cells resulting from RT and further immune stimulation may further directly activate NK cells (5) against the tumor (5a and b). Abbreviations: AnxA5, AnnexinA5; CRT, calreticulin; DC, dendritic cell; Hsp70, heat shock protein 70; Hsp-R, Hsp receptor; HMGB1, high-mobility group box 1 protein; HT, hyperthermia; LDR, low dose radiotherapy; LN, lymph node; NK cell, natural killer cell; RT, radiotherapy with high single-doses applied in tumor therapy; TLR, Toll-like receptor; X-ray, ionizing radiation.

It was shown that higher radiation doses are associated with increased antigen expression (Santin et al., 1997) and induction of necrotic forms of tumor cell death (Mantel et al., 2010). Since necrotic cells have lost their membrane integrity, formerly hidden molecules such as DNA, chaperones, and proteins involved in stabilization of the DNA are released (Beyer et al., 2012). They operate as damage-associated molecular patterns (DAMPs) or alarmins and alert the immune system that “danger” has occurred (Matzinger, 1994). The immunogenicity of necrotic cells is strongly determined by the danger signals high-mobility group box 1 protein (HMGB1) and heat shock protein 70 (Hsp70). However, also CT- and/or RT-induced apoptotic tumor cells can be rendered immunogenic besides exerting their phosphatidylserine (PS)-dependent anti-inflammatory effects (Frey and Gaipl, 2011). The expression of the endoplasmic reticulum (ER)-derived protein calreticulin (CRT) on the tumor cell surface acts as recognition and uptake signal for DCs (Obeid et al., 2007). Further, Hsp70 is released within membranous structures after stressing the cells (Vega et al., 2008). The release of such microvesicles shows strong similarities to those of danger signals. Both events occur during cell death and may lead to stimulation of distinct Toll-like receptors (TLRs) on DCs (Pisetsky et al., 2011). RT-induced necrotic and apoptotic tumor cells may finally stimulate systemic innate (NK cell-mediated) and adaptive (DC and CTL-mediated) immunity against the tumor (Figure 1). Immunogenic tumor cell death induced by RT alone or in combination with further immune stimulation is one elicitor of abscopal anti-tumor responses (summarized in Frey et al., 2012).

### Abscopal anti-tumor effects

This conclusion is supported by clinical observations showing that RT achieves not only local tumor control by stopping the proliferation of and destroying the tumor cells directly at the irradiated site, but additionally results in indirect anticancer effects in non-irradiated areas of the patients. Abscopal effects in the clinics have been observed for various cancer types including hepatocellular carcinoma (Ohba et al., 1998; Okuma et al., 2011), chronic lymphocytic leukemia (Sham, 1995), renal cell carcinoma (Wersall et al., 2006), malignant lymphomas (Nobler, 1969; Antoniades et al., 1977), and melanomas (Kingsley, 1975; Postow et al., 2012).

The phenomenon “abscopal effect” or “distant bystander effect” was originally described by Mole (1953) and the term comes from the latin “ab-” (position away from) and “scopus” (mark or target). Mole defined it “at a distance from the irradiated volume, but within the same organism.” In contrast to the radiation-induced bystander effect, which is mediated via cell-to-cell gap junctions (Azzam et al., 2001) or by secreted soluble factors (TGFβ, NO; Iyer et al., 2000) of irradiated cells that thereby communicate with non-irradiated neighboring (bystander) cells, the abscopal effect is an indirect and systemic effect in non-irradiated areas distant of the irradiated field. Taken together, ionizing radiation induces both local (targeted and bystander effects) and systemic effects (abscopal effects) in cancer patients. However, the cellular and molecular mechanisms of abscopal effects still remain to be clarified.

Various preclinical and clinical studies sustain the assumption that a spontaneous regression of tumors, metastases, or enlarged LN outside of the irradiated field is mediated by the immune system. Konoeda observed an abscopal effect in metastatic LN of breast carcinoma in 15 out of 42 patients. The effect was most frequently noticed when infiltrating CD4+ and CD8+ T cells were present around degenerated tumor cells of the irradiated primary tumor (Konoeda, 1990). Spontaneous regression of a relapsed nodular lesion in a patient with NK cell lymphoma without any treatment was documented by Isobe et al. (2009) after massive infiltration of CD8+ CTLs in the relapsed lesion. Interestingly, the patient was initially treated with radio- and chemotherapy against an eyelid tumor. The susceptibility of tumor cells to CTL-mediated lysis may result from the RT-induced altered tumor cell phenotype associated with increased expression of MHC class I molecules on the surface and an increased intracellular peptide pool (Reits et al., 2006). Demaria et al. (2004) have actually demonstrated in a mouse model of mammary carcinoma that the systemic anti-tumor effect is mediated by the immune system. T cells are required for distant tumor inhibition after combined therapy of the primary tumor with RT and the DC growth factor Fms-like tyrosine kinase receptor 3 ligand (Flt3-L). They concluded that RT alone is a poor inducer of abscopal effects, but that combinations with further immune stimulants are more effective. In their preclinical examinations, the Flt3-L increased the number of DCs at the tumor site. There, DCs assimilate tumor antigens for tumor peptide (cross)-presentation. Maturation signals for DCs are delivered in form of cytokines or other inflammatory stimuli released by the damaged cancer cells resulting after RT (Demaria et al., 2004). In summary, local RT damages tumor cells and generates large amounts of tumor antigens in apoptotic and necrotic tumor cells as well as cellular debris, which, either alone or together, provide immune stimulatory signals for DCs (Figure 1). The latter mature and migrate to draining LN, where they present tumor peptide antigens to naïve T cells. RT further increases the number of interferon (INF)-γ producing T cells in draining LN (Lugade et al., 2005). Despite this, Kim and co-workers demonstrated, in accord with Demaria et al. that conventional RT alone is not sufficient to eliminate tumor masses distant from the irradiated site. The reason could be an inadequate antigen presentation in LN which they overcome by injection of DCs into the irradiated tumor tissue (Kim et al., 2004). Anti-tumor immunity can further be potentiated with an additional administration of the DC danger/maturation signals LPS or TNFα.

The studies outlined above indicate that a proper DC maturation and activation is essential for the induction of an effective anti-tumor T cell response. Apetoh and colleagues identified the TLR4 as one crucial receptor on DCs stimulating the cross-priming of CD8+ CTLs. In addition to LPS, TLR4 also recognizes HMGB1, a nuclear protein passively released as danger signal by late apoptotic or necrotic cells (Apetoh et al., 2007). Using a similar experimental design as Kim et al. (2004), Akutsu et al. (2007) identified the heat shock protein gp96 as a target molecule involved in the abscopal effect. Radiation-induced gp96 is capable to activate DCs via TLR2 and TLR4 (Vabulas et al., 2002). Based on the studies of Lee et al. (2009), Shiraishi et al. (2008), and Dewan et al. (2009), Takeshima et al. showed that CD8+ T cells play a major role in growth inhibition of non-irradiated tumors in combining Th1 cell therapy with local RT. This combination of RT with immune therapy did not only induce the generation of tumor-specific CTLs at the primary tumor site and complete eradication of the tumor, but also prevented the outgrowth of distal tumors (Takeshima et al., 2010).

Likewise combinations of RT with cytokine therapy have the potential to control metastases. The local and systemic effect of ECI301, a human macrophage inflammatory protein-1 alpha variant, in combination with RT was investigated by Shiraishi and colleagues. Their results indicate that the combined therapy reduces the primary tumor growth at the irradiated site and that of distal, non-irradiated tumors. This abscopal effect was dependent of CD8+ lymphocytes, CD4+ lymphocytes, and NK1.1 cells, but independent of the tumor-type and genetic background (Shiraishi et al., 2008). An additional administration of Interleukin-2 (IL-2) to RT also results in a better local tumor control and regression of the not irradiated tumor within the same mouse (Everse et al., 1997; Jurgenliemk-Schulz et al., 1997). Others demonstrated an abscopal effect after manipulation of tumor cells with transgenes expressing several cytokines such as IL-2 (Kwong et al., 1997) or Flt3-L (Dong et al., 2003).

Combinations of RT with antibodies blocking inhibitory negative regulatory molecules on T cells, such as the monoclonal antibody ipilimumab against CTLA-4, are promising to induce systemic anti-tumor immune responses (Dewan et al., 2009). Ipilimumab was approved by the FDA (U.S. Food and Drug Administration) in 2011. In two randomized phase 3 trials, an overall survival benefit in patients with metastatic melanoma was observed (Hodi et al., 2010; Robert et al., 2011). However, autoimmune reactions have to be kept into mind as possible severe side effect of such combined therapies (Mellman et al., 2011). Table 1 summarizes the literature about abscopal effects observed in preclinical and clinical studies after RT and/or immune therapy. It has to be stressed that until today only one major hint for a molecular mechanism for RT-induced abscopal effects has been suggested. Camphausen and co-workers showed that p53 and downstream signals are key mediators of this process. In contrast to the studies mentioned above, a dose-dependent abscopal effect induced by RT alone was observed in wild-type but not in p53 knockout mice (Camphausen et al., 2003).

**Table 1:** **Abscopal anti-tumor effects observed in preclinical and clinical studies after RT and/or immune therapy**.

Tumor-type	Treatment	Abscopal effect	Mediator of abscopal effect	Reference
**CLINICAL REPORTS**				
Hepatocellular carcinoma	RT of thoracic vertebral bone metastases, *dose:* 36 Gy	Regression of primary tumor	TNF-alpha	Ohba et al. (1998)
Hepatocellular carcinoma	RT of mediastinum, *dose:* 27 × 2.25 Gy	Regression of lung metastases		Okuma et al. (2011)
Renal cell carcinoma	RT of primary tumor, *dose:* 12 × 8Gy	Regression of enlarged lymph nodes and lung lesions		Wersall et al. (2006)
Mammary carcinoma	RT of primary tumor	Regression of metastatic lymph nodes	CD8+ and CD4+ T cells	Konoeda (1990)
NK-ENKL	RT of eyelid tumor	Regression of NK cell lymphoma	CD8+ T cells	Isobe et al. (2009)
**MOUSE MODELS**				
Mammary carcinoma (67NR)	RT of primary tumor, *dose:* 2, 6 Gy, i.p. administration of Flt3-L (10×)	Growth delay of non-irradiated 67NR tumors, tumor-type dependent	DCs, T cells, RT + Flt3-L	Demaria et al. (2004)
Squamous cell carcinoma (SCCVII)	RT of primary tumor, *dose:* 3 × 4 Gy, i.t. administration of DCs	Growth inhibition of non-treated tumor	DC, gp96, RT+ i.t. DCs	Akutsu et al. (2007)
Mammary carcinoma (4T1)	RT of primary tumor, *dose:* 1 × 20 Gy	Elimination of lung metastases	CD8+ T cells	Lee et al. (2009)
Adeno-carcinoma (Colon26)	RT of primary tumor, *dose:* 6Gy, i.v. administration of ECI301	Growth inhibition of non-irradiated tumor, tumor-type independent	CD8+ and CD4+ T cells, NK1.1 cells, IFN-γ, RT + ECI301	Shiraishi et al. (2008)
Mammary carcinoma (TSA), mouse colon carcinoma (MCA38)	RT of primary tumor, *dose:* 20 Gy, 3 × 8 Gy, 5 × 6 Gy, administration of anti-CTLA-4 mAb	Growth inhibition of non-irradiated tumor	CD8+ and CD4+ T cells, IFN-γ, Fractionated RT + anti-CTLA-4 mAb	Dewan et al. (2009)
Lymphoma (EG7)	RT and Th1 cell therapy	Growth inhibition of non-irradiated tumor	CD8+ T cells	Takeshima et al. (2010)
Lymphoma (SL2), mammary carcinoma (M8013)	RT of primary tumor, *dose:* 20 Gy, 10 × 2.5 Gy, peritumoral administration of rIL-2	Regression of non-irradiated tumor	Radio-immunotherapy	Jurgenliemk-Schulz et al. (1997)
Lymphoma (SL2)	RT of primary tumor, *dose:* 20 Gy, 10 × 2.5 Gy, peritumoral administration of rIL-2	Regression of non-irradiated tumor	Radio-immunotherapy	Everse et al. (1997)
Lewis lung carcinoma (LL2)	LL2 transfected with viral vector expressing IL-2	Regression of hepatic lung cancer metastases		Kwong et al. (1997)
Squamous cell carcinoma (B4B8)	B4B8 transfected with plasmid encoding Flt3-L	Growth inhibition of non-treated tumor		Dong et al. (2003)
Lewis lung carcinoma (LLC), fibrosarcoma (T241)	RT of normal tissue, *dose:* 5 × 10 Gy, 12 × 2 Gy	Growth inhibition of non-irradiated tumor, tumor-type independent	p53	Camphausen et al. (2003)

In conclusion, cancer treatments which activate enough DCs and eventually the adaptive immune system and further directly cells of the innate immune system (see below) are promising approaches to improve the eradication of primary tumors and metastases (Figure 1). An optimized radiation regimen combined with immune therapy makes indeed anticancer therapies more efficient.

## Anti-Tumor Immunity Induced by Combination of RT with Further Immune Stimulation by AnnexinA5 or Heat

Additional approaches to induce a CTL-mediated tumor cell killing are based on the *in vivo* activation of DCs, which should take up tumor antigens and consecutively present tumor peptides to T cells to achieve co-stimulation. However, macrophages recognize and phagocytose dying tumor cells swiftly and silently and thereby remove tumor antigens (Gaipl et al., 2007). They are recruited by find-me signals such as lysophosphatidylcholine (Lauber et al., 2003) secreted by RT-induced apoptotic cells. The latter may even cause caspase 3-dependent tumor cell repopulation by generating potent growth-stimulating signals (Huang et al., 2011). Moreover, an anti-inflammatory milieu results from the clearance of those apoptotic cells by macrophages (Lauber et al., 2011). Since DCs and macrophages partly utilize different clearance mechanisms (Hoves et al., 2011), one possibility to enable enhanced access of DCs to RT-induced apoptotic and necrotic tumor cells is to block their clearance by macrophages with the PS-binding protein AnnexinA5 (AnxA5; Bondanza et al., 2004; Frey et al., 2009). The growth of syngeneic tumors is significantly retarded by a single injection of AnxA5 around the tumor. Combination of RT with AnxA5 resulted in the most effective inhibition of tumor growth (Frey et al., 2009). *In vivo* experiments with immune competent mice bearing syngeneic tumors have proven that AnxA5 increases the immunogenicity of tumor cells. The injection of irradiated tumor cells pre-incubated with AnxA5 cured established tumors in about 50% of the animals, while the injection of irradiated tumor cells only resulted in less than 10% of tumor free mice (Bondanza et al., 2004). Since RT induces tumor cell death and thereby the exposure of PS on dying tumor cells and on tumor blood vessels, it represents an adequate combination partner with PS-targeting agents such as AnxA5, and monoclonal antibodies such as the murine 2aG4 antibody (He et al., 2007). Phase I and II clinical trials with bavituximab, the human analog to 2aG4, in combination with standard therapies for the treatment of solid tumors are currently performed (Derose et al., 2011). In preclinical rat models, combination of PS-targeting with RT resulted in long-term anti-tumor immunity even against glioblastoma in over 10% of the animals (He et al., 2009). Recently Riedl et al. (2011) showed that PS is also exposed by non-dying tumor cells, preferentially in metastases. Targeting of PS on therapy-induced dying and on viable metastatic cells could therefore both lead to efficient anti-tumor immune responses by promoting uptake of the tumor cells by DCs, to mention here one of multiple possible modes of action resulting from the shielding of PS (summarized in Frey et al., 2012).

Cross-presentation of tumor peptides by DCs requires antigen uptake and additionally a maturation signal for DCs to avoid tolerance induction. The maturation of immune stimulatory DCs is stimulated by necrotic tumor cells (Sauter et al., 2000). Extracellular heat shock proteins act as immune activating danger signals and fulfill both functions: they are means of transport for tumor antigens and elicitors of DC maturation (Basu et al., 2000; Somersan et al., 2001). Appropriately, DCs pulsed with tumor cells that have been heat-shocked mediated a significant enhanced cellular T cell cytotoxicity response against the tumor cells compared to pulsed DCs with lysates of non-heat-shocked cells (Schueller et al., 2003). In addition, combination of RT with HT increased the amount of released danger signals such as HMGB1 and Hsp70 and further fosters the maturation of DCs (Schildkopf et al., 2009a,b, 2011; Figure 1). Future research should focus on preclinical *in vivo* models to examine which immune cell subsets get recruited into the tumor after local treatment with RT plus HT and under which treatment combinations the maximum DC-mediated and MHC-dependent CTL activation takes place. Chen et al. (2009) have already demonstrated in mouse models that heat-stressed tumor cells are capable of initiating anti-tumor immune responses by inducing activation of DCs. Immunological back-up or parallel mechanisms for tumor cell killing should be considered, since tumor cells often shed MHC I molecules. The exposure of Hsp70 on the tumor cell surfaces serves as a recognition signal for activated NK cells (Stangl et al., 2008). NK cells are activated against the tumor when tumor cells have shed MHC class I molecules to escape killing by CTLs, since the inhibitory receptors of NK cells are no longer triggered by MHC I molecules. Following HT treatment, NK cells have been found to be enriched at the tumor site (Burd et al., 1998), showing that innate immune responses also contribute to the fight against the tumor.

## Outlook

We have outlined that immunogenic tumor cell death forms are induced by RT with additional immune stimulation. Figure 1 schematically depicts how ionizing radiation (X-ray) could stimulate innate and adaptive immune responses against the irradiated tumor as well as against non-irradiated ones (abscopal effects). The current knowledge suggests that induction of tumor cell necrosis including necroptosis (Vanlangenakker et al., 2012) and apoptosis by RT and further immune stimulators is most beneficial for the induction of a specific and long-lasting anti-tumor immunity (Kepp et al., 2009). Since the phenotype of the individual tumor of a distinct patient is modified by RT, the best possible personalized treatment approach is realized. Although tumor regression is often the main indicator for a successful therapy, this may not always translate into improved survival rates. Since the immune system needs time to act, the success of immune therapies is often observed at later time points after the treatment and connected to long-term survival rates, as shown in clinical trials with the CTLA-4-blocking antibody ipilimumab (Mellman et al., 2011). Since *in vivo* assays revealed that DCs require approximately 48 h for migration into the tumor, tumor antigen uptake, maturation, and consecutive migration to the sentinel lymph node (Wheeler et al., 2004), innovative irradiation schemes could be that hypofractionated ones expand the days where no irradiation takes place. This could avoid that activated DCs in the tumor microenvironment are killed by RT. Further studies are needed to document which combinations of RT and immune therapies and which time windows of combination are most effective to induce specific and long-lasting anti-tumor immune responses.

## Conflict of Interest Statement

The authors declare that the research was conducted in the absence of any commercial or financial relationships that could be construed as a potential conflict of interest.
